# Hochaltrigkeit und Klimawandel – ein neues Forschungsfeld

**DOI:** 10.1007/s00103-026-04281-z

**Published:** 2026-08-11

**Authors:** Grit Höppner, Martina Brandt

**Affiliations:** 1 Fachbereich Sozialwesen, Katholische Hochschule Nordrhein-Westfalen, Piusallee 89, 48147 Münster, Deutschland; 2 Fakultät Sozialwissenschaften, Sozialstruktur und Soziologie Alternder Gesellschaften, Technische Universität Dortmund, Dortmund, Deutschland

**Keywords:** Extremwetterereignisse, Alter, Anpassungsstrategien, Umweltbewusstsein, Nachhaltigkeit, Extreme weather events, Old age, Coping strategies, Environmental consciousness, Sustainability

## Abstract

Der Klimawandel stellt das menschliche Zusammenleben auf regionaler, nationaler und globaler Ebene vor große Herausforderungen. Die damit einhergehenden Extremwetterereignisse betreffen gerade in schnell alternden Gesellschaften wie Deutschland zunehmend ältere und hochaltrige Menschen. Im Diskurs über Anpassungsstrategien und umweltbewusstes Handeln wird die Diversität des Alter(n)s jedoch bislang nicht systematisch untersucht. Insbesondere Hochaltrige, die auf Unterstützung oder Pflege angewiesen sind, geraten sowohl in der Gerontologie als auch in der Umwelt- und Nachhaltigkeitsforschung häufig aus dem Blick.

Ziel des Beitrags ist es, das an der Schnittstelle dieser beiden Forschungsbereiche entstehende neue Forschungsfeld zu beleuchten. Hierzu stellen wir zunächst den aktuellen Forschungsstand in der Alter(n)sforschung sowie der Umwelt- und Nachhaltigkeitsforschung dar und gehen anschließend auf die bisherigen Erkenntnisse zu ihren Verbindungen ein. Dabei zeigen wir bestehende Forschungslücken hinsichtlich der Anpassungsstrategien Hochaltriger sowie ihrer Möglichkeiten zu umweltbewusstem Handeln auf und skizzieren Perspektiven für die zukünftige theoretische und empirische Weiterentwicklung dieses Forschungsfeldes.

## Abstract (en)

Climate change poses significant challenges to people and societies at regional, national, and global levels. The associated extreme weather events increasingly affect older and very old people, particularly in rapidly ageing societies such as Germany. However, the diversity of ageing is not systematically considered in the discourse on adaptation strategies and environmentally conscious behavior. In particular, the oldest old who depend on care are often overlooked in both gerontology and environmental research.

This article aims to shed light on the research field emerging at this intersection. First, we present the current state of research in gerontology and in environmental and sustainability research, before we outline their relation. Then, we identify gaps in the literature concerning adaptation strategies for the oldest old and their opportunities for environmentally conscious behavior. We conclude with a look at future theoretical and empirical development opportunities and necessities in this field.

## Einleitung

Der Klimawandel bringt tiefgehende Veränderungen der Lebensumwelt mit sich, die das menschliche Zusammenleben auf regionaler, nationaler und globaler Ebene vor große Herausforderungen stellen. Klimatische Veränderungen, die sich in Extremwetterereignissen äußern, betreffen gerade in schnell alternden Gesellschaften wie Deutschland immer mehr Ältere und Hochaltrige mit Vorerkrankungen und Mobilitätseinschränkungen. Im Diskurs über mögliche Anpassungsstrategien und umweltbewusstes Handeln wird die Diversität des Alter(n)s jedoch bislang nicht systematisch berücksichtigt. Insbesondere die „alten Alten“ [1] bzw. „Hochaltrigen“ (je Kontext und Studie 75+/80+/85+), die aufgrund von körperlichen und kognitiven Einschränkungen auf Unterstützung oder Pflege angewiesen sind, geraten sowohl in der Gerontologie als auch in der Umwelt- und Nachhaltigkeitsforschung aus dem Blick. So entsteht in Zeiten einer schnell wachsenden Zahl Hochaltriger, die immer mehr Hitzewellen, Trockenperioden, Waldbränden, extremer Kälte, Stürmen und Starkregen ausgesetzt sein werden, ein zentrales neues Forschungsfeld, das wir im Folgenden ausleuchten wollen.

Wir zeigen zunächst den Stand der Forschung in der Alter(n)sforschung und Umwelt- und Nachhaltigkeitsforschung und, soweit vorhanden, an deren Schnittstelle auf, bevor wir näher auf die Forschungslücken im Bereich der Anpassungsstrategien Hochaltriger sowie ihrer Möglichkeiten umweltbewussten Handelns eingehen. Abschließend zeigen wir zukünftige Weiterentwicklungsmöglichkeiten und -notwendigkeiten in Theorie und empirischer Forschung auf.

## Forschungsstand in der Alter(n)sforschung sowie der Umwelt- und Nachhaltigkeitsforschung

Die Bedrohung durch den Klimawandel steigt stetig: Berichte wie der 2023 publizierte „Lancet Countdown on health and climate change“ belegen, dass im Jahr 2022 weltweit Temperaturrekorde gebrochen wurden [2] – ein Trend, der sich fortsetzen wird. Im Deutschen Alterssurvey im Jahr 2023 gibt gut jede 4. Person zwischen 43 und 90 Jahren an, dass der Klimawandel eine große Bedrohung für sie darstellt, knapp die Hälfte der Befragten schätzt die Bedrohung im mittleren Bereich ein [3]. Weiterhin wurden für Menschen mit Vorerkrankungen und Menschen mit niedrigem Einkommen erhöhte Sterblichkeitsrisiken nachgewiesen [4–6]. Zusätzlich zu sozioökonomischen Ungleichheiten im Klimawandel zeigen sich soziale Spaltungen und Generationenkonflikte, die unter anderem durch (altersassoziierte) Verantwortungszuschreibungen entstehen [7].

Studien zum Klimawandel, zu Umweltbelastungen und zu Extremwetterereignissen nehmen jüngst deutlich zu, ohne aber bisher die Vielfalt der Lebenssituationen Älterer und insbesondere die spezifische Gruppe der hochaltrigen Menschen (hier: ab 80 Jahren) explizit und systematisch zu berücksichtigen. Dabei zeigt nicht erst der jüngst erschienene 9. Altersbericht der Bundesregierung „Alt werden in Deutschland – Vielfalt der Potenziale und Ungleichheit der Teilhabechancen“ deutlich, wie vielfältig Alter und Altern sind und wie sich damit verbundene soziale Ungleichheiten überlagern und verstärken können [8]. Dass Alter und Altern selbst als Ungleichheitskategorien bedeutsam werden [9], zeigt sich in neueren Altersbildern, die sich zumeist aber vor allem auf die sogenannten jungen Alten beziehen und „aktives“, „produktives“, „erfolgreiches“ Altern fordern (kritisch hierzu z. B. [1]).

Dabei lebten im Jahr 2024 schon rund 6 Mio. Menschen in Deutschland, die 80 Jahre und älter waren [10]. Mit zunehmendem Alter steigt der Anteil der Pflegebedürftigen an – im Jahr 2019 waren 26,4 % der 80- bis 85-Jährigen, 49,4 % der 85- bis 90-Jährigen und 76,3 % der über 90-Jährigen pflegebedürftig [11]. 86 % der Pflegebedürftigen werden zu Hause versorgt (67 % überwiegend durch Angehörige bzw. 19 % mit ambulanter Unterstützung), 14 % im Heim [12]. Bei ihnen verschränken sich mehrere Risikofaktoren wie geringere gesundheitliche und ökonomische Ressourcen systematisch – mit stark steigender Tendenz (siehe z. B. [8]). Gerade diese hochaltrigen Menschen sind gesundheitlich durch Extremwetterereignisse bedroht; ihre Bedarfe, Bedürfnisse und Möglichkeiten unterscheiden sich von denen jüngerer Menschen [13–15].

Ein Scoping-Review der Fachliteratur mit Schwerpunkt auf den letzten 15 Jahren im europäischen und angloamerikanischen Raum verdeutlicht, dass der Fokus der Forschung vor allem auf den Konsequenzen von Extremwetterereignissen für das Wohlbefinden, die körperliche und mentale Gesundheit sowie die Mortalität von Menschen in der 2. Lebenshälfte liegt [16]. Dabei überwiegen Studien zu den Auswirkungen von Hitzewellen (u. a. [6, 17–23]), seltener wurden bisher die Auswirkungen von Kälte (u. a. [24–26]) und Starkregen bzw. Überschwemmungen (u. a. [14]) untersucht.

Eine Reihe von Studien belegt eindrücklich die negativen Einflüsse von Hitze auf Gesundheit auch in Deutschland: So wurde für die Hitzewelle im Jahr 2015 in Frankfurt am Main ein Zusammenhang zwischen extremer Hitze und den Einweisungen ins Krankenhaus nachgewiesen, mit einem Anstieg um 22 % [27]. Eine vom Robert Koch-Institut (RKI) durchgeführte Studie zeigt, dass es in den Sommern 2023 und 2024 jeweils ca. 3000 hitzebedingte Todesfälle vorrangig im urbanen Raum in West- und Süddeutschland gab [4]. In der kürzlich veröffentlichten Studie von Masselot et al. [28] wird prognostiziert, dass zukünftig der Anteil der hitzebedingten Todesfälle in weiten Teilen Europas weiter steigen wird, darunter wortwörtliche „Hotspots“ auch in Süddeutschland. Menschen in der 2. Lebenshälfte sind somit heute häufiger Hitzewellen ausgesetzt, als es Gleichaltrige bisher waren; und zugleich haben Menschen über 75 Jahren mit altersassoziierten Erkrankungen wie Demenz, Herz-Kreislauf- oder Lungenerkrankungen ein höheres Hitzetodrisiko als jüngere Altersgruppen [4, 14, 29].

Gerade in der Umwelt- und Nachhaltigkeitsforschung wurden Hochaltrigkeit sowie deren Verknüpfung mit weiteren Ungleichheitsdimensionen wie Geschlecht, sozioökonomischem Hintergrund und kultureller Herkunft im Kontext des Klimawandels bislang nicht systematisch untersucht. So werden beispielsweise in der Studie „Repräsentativumfrage zum Umweltbewusstsein und Umweltverhalten im Jahr 2020“ des deutschen Umweltbundesamtes [30] Menschen in der 2. Lebenshälfte in die Cluster „60 bis 69 Jahre“ und „70 Jahre und älter“ eingeteilt und erste Publikationen aus dem Deutschen Umweltpanel GLEN differenzieren die Ergebnisse im Alter nach 50–59 und 60+ [31]. Das Bild setzt sich auf europäischer Ebene fort: In der Studie „Special Eurobarometer 459“ [32] werden Menschen in der 2. Lebenshälfte als „55+“ geclustert und im „EIB Climate Survey“ [33] als „65 years old and over“. Auf Basis des Sozio-oekonomischen Panel (SOEP) werden Menschen in der 2. Lebenshälfte in der Untersuchung des Zusammenhangs von Einkommen und Hitzeanfälligkeit als eine von mehreren Gruppen unter dem Sammelbegriff „vulnerable households“ subsumiert (u. a. [34]). Selbst in Studien, die das Konzept der Umwelt- und Klimagerechtigkeit nutzen, um aus einer intersektionalen Perspektive die Bedeutung des Zusammenwirkens verschiedener Ungleichheitsdimensionen zu beleuchten und Klimaschutzmaßnahmen nicht losgelöst von Fragen der sozialen Gerechtigkeit zu betrachten [35], wird Hochaltrigkeit nicht explizit berücksichtigt (als Überblick siehe z. B. [36]). Das liegt vermutlich unter anderem auch an geringen Fallzahlen und der Selektivität der Stichproben im sehr hohen Alter in vielen Interviewstudien (s. a. [37]), die einen verstärkten Einsatz von kostspieligen Interviewtechniken (Proxy-Interviews, Einbeziehung von Institutionen etc.) erfordern.

Auch wenn es um die Frage geht, wie mit den neuen klimatischen Herausforderungen umzugehen ist, stehen Ältere und Hochaltrige selten im Fokus: Mit Blick auf (1) Anpassungsstrategien an Extremwetterereignisse bis hin zu (2) umweltbewusstem und nachhaltigem Handeln, die wir im folgenden Abschnitt beleuchten werden, wird selbst im gerontologischen Fachdiskurs häufig ein homogenes Altersbild vermittelt und die Lebensphase „Alter“ nicht differenziert betrachtet. Hier liegt zwar mit der Studie „Hohes Alter in Deutschland“ [38] nun eine repräsentative Erfassung der Lebenssituationen und Lebensqualität von hochaltrigen Menschen für den gesamtdeutschen Raum vor, die bisherige Altersstudien wie der „Deutsche Alterssurvey“ oder auch der „Survey of Health, Ageing and Retirement in Europe“ nicht leisten können. Die Fragen in Bezug auf den Klimawandel beschränken sich jedoch auf das individuelle Wertesystem hochaltriger Menschen: Für 83 % der Befragten ist es wichtig, sich um Natur und Umwelt zu kümmern. Zudem äußern die Befragten Wünsche für einen gelingenden Klima- und Umweltschutz, die sich vor allem an die Politik richten [39]. Insgesamt zeigen sich also dynamische Entwicklungen der Forschung mit ganz neuen Studien und Daten zu Umweltfragen (Deutsches Umweltpanel GLEN) sowie zur Hochaltrigkeit (Hohes Alter in Deutschland: D 80+) – die Verschränkung der Felder ist jedoch noch eine Zukunftsaufgabe.

## Bisherige Erkenntnisse an der Schnittstelle von Alter(n)sforschung und Umwelt- und Nachhaltigkeitsforschung

### Anpassungsstrategien Hochaltriger

Die Anpassungsfähigkeit der Menschen ist ein zentraler Aspekt im Umgang mit klimatischen Veränderungen [40]. Gerontologische Studien in diesem Bereich fokussieren insbesondere auf den Aspekt extreme Hitze [16] und beleuchten im Wesentlichen 3 Anpassungsdimensionen: Alltagsroutinen, soziale Netzwerke und Umgebungsbedingungen.

#### Alltagsroutinen.

Vorrangig spielen in der Forschung kurzfristige Bewältigungsstrategien zur Minimierung von individuellen Risikofaktoren im Alltag eine Rolle. Dabei zeigen internationale qualitative Studien, dass gesunde Menschen in der 2. Lebenshälfte über eine hohe Anpassungsfähigkeit an Extremwetterereignisse verfügen, weil sie nicht mehr den Zwängen des Arbeitslebens unterliegen und ihre täglichen Gewohnheiten leichter anpassen können [22]. Hierzu zählen Veränderungen von Alltagsroutinen, wie das Verschieben des Wocheneinkaufs, die Nutzung einer Klimaanlage, das Aufsuchen klimatisierter Räume wie Supermärkte [41], das Abdunkeln des Wohnraums, das Tragen von leichter Kleidung sowie Anpassungen der Ernährung und eine ausreichende Flüssigkeitsaufnahme [42].

Studien aus Deutschland, die dies quantitativ vertiefen, kommen zu unterschiedlichen Ergebnissen: In einer Studie von Conrad und Penger [17] in Stuttgart wurden 211 Menschen zwischen 65 und 92 Jahren (der genaue Anteil der Hochaltrigen ist nicht ersichtlich) mit vergleichsweise hohen sozioökonomischen, psychosozialen und gesundheitlichen Ressourcen zum Zusammenhang zwischen Hitze und subjektivem Gesundheitserleben sowie zu Anpassungsstrategien befragt. Die Autorinnen zeigen, dass nicht alle Teilnehmenden gleichermaßen hitzegefährdet sind. Hitzevulnerable Personen sind sich der Hitzebelastung jedoch bewusst, passen ihr Mobilitätsverhalten bei Hitze an und vermeiden physische Anstrengung (siehe auch [43, 44]).

Pfaffenbach und Siuda [45], die in Aachen 2121 Menschen ab 50 Jahren zur wahrgenommenen Hitzebelastung und zu Strategien im Umgang mit Hitzephasen schriftlich befragten, zeigen jedoch, dass knapp 30 % der 190 Hochaltrigen, die an der Studie teilgenommen haben, Hitze als (sehr) starke Belastung empfinden, während etwa 20 % der Befragten unter 80 Jahren diese Angabe machte. Die Autorinnen begründen die höhere Hitzeempfindlichkeit von Hochaltrigen im Vergleich zu jüngeren Befragten nicht nur mit dem höheren Alter, sondern auch mit geringer ausgeprägten Anpassungsstrategien. Zwar suchen Hochaltrige bei hitzebedingten gesundheitlichen Problemen häufiger eine Ärztin oder einen Arzt auf und sind daher über Risiken und angemessenes Verhalten bei Hitze etwas aufgeklärter als jüngere Studienteilnehmende. Anders als in der Studie von Conrad und Penger [17] ändern Hochaltrige an heißen Sommertagen laut dieser Studie ihr Verhalten aber nur punktuell (mehr ausruhen, häufiger zu Hause bleiben) oder nicht (seltener als jüngere Befragte trinken sie in Hitzephasen mehr).

Eine weitere wenig erforschte Gruppe sind Menschen, die auf Pflege angewiesen oder bettlägerig sind und kaum Kapazitäten haben, bei extremer Hitze selbstständig Anpassungsstrategien zu ergreifen [22, 23]. Eine weitere Forschungslücke betrifft die Potenziale der (allgemein-)medizinischen Versorgung im Umgang mit Extremwetterereignissen. Vor dem Hintergrund der derzeit entwickelten Leitlinie zur klimasensiblen Gesundheitsberatung für allgemeinmedizinische Praxen^1^ ist bislang noch unzureichend erforscht, welchen Beitrag eine solche Beratung insbesondere für hochaltrige Menschen leisten kann.

#### Soziale Netzwerke.

Unterschiedliche Befunde gibt es auch zur Rolle von sozialen Kontakten und Netzwerken. Einerseits weisen Studien auf die Bedeutung von sozialer Unterstützung für Menschen in der 2. Lebenshälfte während Hitzewellen hin, gerade vor dem Hintergrund der verstärkten sozialen Isolation während Hitzebelastung [41, 46]. Andererseits scheinen insbesondere sozial aktive Menschen die eigene Hitzeanfälligkeit zu unterschätzen, wenn sie in soziale Netzwerke eingebunden sind, die sie als vulnerabler als sich selbst erleben und bewerten [18, 42, 47].

#### Umgebungsbedingungen.

Nicht zuletzt untersuchen gerontologische Studien räumliche und kontextuelle Faktoren, die die Resilienz von Menschen in der 2. Lebenshälfte stärken und dazu beitragen, sich längerfristig vor den Folgen von Extremwetterereignissen zu schützen. Hierzu zählen im stärker hitzebelasteten urbanen Raum vor allem bauliche Veränderungen zur Kühlung des Wohnraums, das Vermeiden des Wohnens im Dachgeschoss und der Zugang zu Grünflächen. Knappe finanzielle Ressourcen können jedoch ein Hindernis in der Umsetzung darstellen (u. a. [48, 49]). Es zeigt sich entsprechend, dass in den letzten Jahren verstärkt strukturelle Maßnahmen wie Hitzepläne, Konzepte zu präventiven Maßnahmen (z. B. Baumaßnahmen im öffentlichen Raum) und digitale Warnsysteme für Hitze und Gesundheit erarbeitet wurden [50, 51], um Menschen insbesondere im urbanen Raum und in der stationären Pflege zu schützen (z. B. [52, 53]).

Es sind also nicht allein individuelle Bewältigungsstrategien, sondern auch alters- und geschlechtsbezogene, sozioökonomische und räumlich-kontextuelle Faktoren, die die Anpassungsfähigkeit von Menschen an Extremwetterereignisse beeinflussen [54]. Zugleich zeigen sich auch im Kontext des Klimawandels ausgeprägte soziale Ungleichheiten sowohl hinsichtlich des Ausgesetztseins gegenüber klimabedingten Risiken als auch der Möglichkeiten zu deren Bewältigung: Hochaltrige Menschen mit geringen sozioökonomischen Ressourcen und in der stationären Pflege leiden unverhältnismäßig stark unter den negativen Auswirkungen des Klimawandels, was zu einer weiteren Verschärfung von sozialen Ungleichheiten führt [55, 56]. In Abhängigkeit von ihrer individuellen Konstitution und ihren Lebensverhältnissen verfügen Menschen in der 2. Lebenshälfte über spezifische Kapazitäten, um mit klimabedingten Extremwetterereignissen umzugehen und Gesundheit und Wohlbefinden zu erhalten. Dies gilt auch für (bis dato wenige untersuchte) Kälteereignisse: Auch hier beeinflussen individuelle Ressourcen (u. a. Einkommen), soziale Netzwerke und die Wohnverhältnisse die Anpassungsfähigkeit von Menschen in der 2. Lebenshälfte [26], auch im Hinblick auf steigende Energiekosten bei knappen finanziellen Ressourcen [41].

Insgesamt zeigt sich im Hinblick auf Anpassungsmöglichkeiten: Obwohl Hochaltrige (durchschnittlich) besonders gefährdet sind, wissen wir zu wenig darüber, welche kurz- und längerfristigen Strategien hochaltrige Frauen und Männer mit und ohne Pflegebedarf, unterschiedlicher sozioökonomischer Hintergründe und kultureller Herkunft in unterschiedlichen Wohnsituationen und in unterschiedlichen Regionen Deutschlands entwickelt haben und welche Ressourcen und Kapazitäten ihnen zur Verfügung stehen, um unterschiedliche klimabedingte Extremwetterereignisse zu bewältigen.

### Umweltbewusstes und nachhaltiges Handeln Älterer und Hochaltriger

Die vorgestellten Studien zum Klimawandel und zu Extremwetterereignissen nehmen die Konsequenzen für Menschen in der 2. Lebenshälfte in den Blick und untersuchen, wie diese auf klimatische Veränderungen reagieren. Die entgegengesetzte Perspektive – Handlungsmöglichkeiten zur aktiven Beeinflussung des Klimawandels in Form von umweltbewusstem und nachhaltigem Handeln – wird seltener thematisiert [16]. Ältere wurden bis dato eher für den Klimawandel, denn für seine Bekämpfung verantwortlich gemacht [57].

In Studien zu Handlungsmöglichkeiten Älterer werden diese jedoch als Teil der Lösung von Umweltkrisen konstruiert, wobei zwischen „schwach“ und „stark nachhaltig“ Handelnden unterschieden wird [58, 59]. Als schwach nachhaltig gelten ältere Menschen, die Energie und Wasser sparen, recyceln und weniger reisen [60], als stark nachhaltig solche, die als „Naturliebhaber:innen“ und „Bewahrer:innen des Ökosystems“ durch freiwilliges, zivilgesellschaftliches Engagement im Umweltschutz einen aktiven Beitrag leisten. Ein solches sinnstiftendes gemeinschaftliches Handeln kann, so betont die Forschung, die soziale Integration fördern, die Gemeinschaftsbildung unterstützen sowie Partizipation und Vernetzung mit Akteur:innen aus Zivilgesellschaft, Verwaltung und Politik ermöglichen [61–63].

In der Umwelt- und Nachhaltigkeitsforschung belegen Studien die Bedeutung von Umweltwahrnehmungen und -einstellungen als zentrale Determinanten für Umweltverhalten, etwa für die Reduzierung des individuellen CO_2_-Fußabdrucks beim Lebensmittel- und Energieverbrauch sowie beim Transport (u. a. [64]). Mehrheitlich zeigen die Studien, dass soziodemografische Merkmale wie Alter, Bildung und Geschlecht das Umweltbewusstsein, die Akzeptanz von klimapolitischen Maßnahmen und nachhaltiges Handeln beeinflussen (u. a. [65–67]).

Auch wenn in Studien das Umweltbewusstsein von Menschen in der 2. Lebenshälfte hervorgehoben wird (z. B. [68, 69]), werden Ältere im Allgemeinen und Hochaltrige im Besonderen aber kaum als Adressat:innen von politischen Nachhaltigkeitsprogrammen angesprochen und erreicht. Entsprechend ist auch ihr Potenzial für stark nachhaltiges Handeln vermutlich noch nicht ausgeschöpft [14]. Eine empirisch offene Frage ist insbesondere, inwiefern Hochaltrige umweltbewusst leben und in ihrem Alltag nachhaltig handeln (können und wollen).

## Forschungslücken schließen

Der Überblick über den Stand der Forschung zu Hochaltrigkeit und Klimawandel lässt sich wie folgt bilanzieren: Sowohl in gerontologischen Arbeiten als auch in Studien der Umwelt- und Nachhaltigkeitsforschung wird in der Mehrheit der Arbeiten ein zu homogenes Bild des höheren Lebensalters im Kontext des Klimawandels vermittelt. Ältere und hochaltrige Menschen werden entweder zusammengefasst (z. B. als „65+“), gemeinsam mit Gruppen untersucht, denen gleiche Merkmale zugeschrieben werden (z. B. Vulnerabilität), oder sie werden in der Auswertung nicht explizit berücksichtigt. Ausgeblendet werden auf diese Weise (1) hochaltrige Menschen als eine besonders vulnerable Zielgruppe mit spezifischen Bedarfen und somit (2) auch die Heterogenität des Alters [9]. Nicht ausreichend untersucht sind dadurch auch (3) ungleiche Lebensverhältnisse, die Anpassungsstrategien speziell von hochaltrigen Menschen an klimatische Veränderungen beeinflussen, und (4) ihre subjektiv erlebten Teilhabemöglichkeiten, die ein nachhaltiges Alter und Altern prägen. Neue Hochaltrigkeitsstudien [70] beschäftigen sich zudem nur punktuell mit Auswirkungen des Klimawandels auf hochaltrige Menschen.

Insgesamt überwiegen in der Forschung bisher Studien zu den Auswirkungen von Hitzewellen. Demzufolge wissen wir zu wenig darüber, mit welchen veränderten Kontextbedingungen aufgrund des Klimawandels hochaltrige Menschen in ihrem Alltag konkret umgehen müssen und welche Routinen bzw. welche Anpassungs- und Bewältigungsstrategien sie entwickeln, um auf unterschiedliche klimatische Veränderungen und Extremwetterereignisse zu reagieren. Unbeantwortet ist so auch die Frage, inwiefern hochaltrige Menschen umweltbewusst und nachhaltig handeln.

### Theoretische Modelle erweitern.

Zur Schließung dieser eklatanten, im doppelten Sinne intersektionalen Forschungslücken müssen theoretische Modelle insbesondere mit Blick auf Diversität und Ungleichheiten in der Lebensphase Alter angepasst und erweitert werden, um methodische Anforderungen an die Erforschung sich überlagernder Ungleichheiten theoretisch zu begründen, Messinstrumente für zukünftige Studien weiterzuentwickeln und empirische Befunde angemessen einzuordnen (siehe auch [71]). Auf Basis des vorliegenden Überblicks über die Alter(n)s- und Umwelt- und Nachhaltigkeitsforschung lässt sich ein Modell des nachhaltigen Alters und Alterns ableiten [16], das um Hochaltrigkeit erweitert werden kann und muss.

**Abb. 1:**
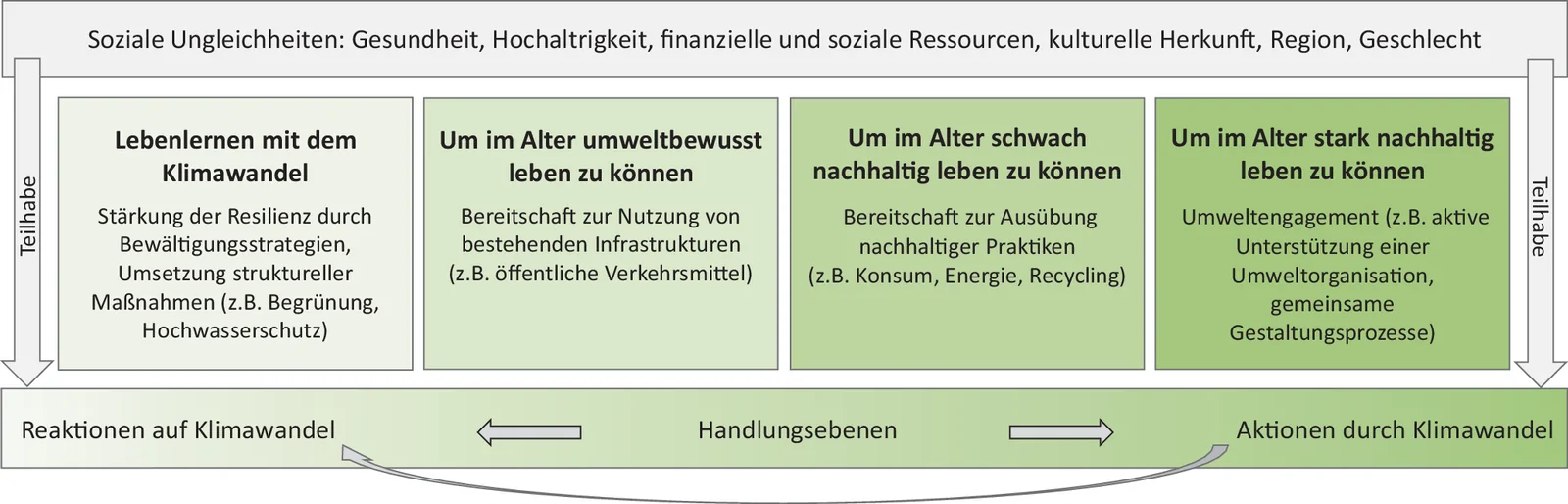
Modell des nachhaltigen Alters und Alterns im Kontext des Klimawandels. Quelle: Weiterentwicklung aus [16]

Das Modell des nachhaltigen Alters und Alterns im Kontext des Klimawandels (Abb. 1) zeigt, dass Alterskonstruktionen, die Alter und Altern über biologische Gegebenheiten hinaus als sozial konstruierte Phänomene beschreiben, im Kontext des Klimawandels vielfältiger sind und werden müssen, als dies die Fachliteratur mit dem Fokus auf Konsequenzen für die Gesundheit nahelegt, sowohl im Hinblick auf soziale Ungleichheiten als auch auf die Handlungsebenen. Das Modell umfasst deshalb sowohl unterschiedliche Ungleichheitsdimensionen als auch ein Spektrum an Handlungen, wobei der linke Pol Reaktionen auf den Klimawandel und der rechte Pol Aktionen, die durch den Klimawandel bedingt sind, markiert. Ältere und Hochaltrige sind nicht nur vulnerabel und dem Klimawandel weitestgehend schutzlos ausgeliefert, sondern sie verfügen über die Fähigkeit zur Anpassung an die Veränderungen und können umweltbewusst und unterschiedlich stark nachhaltig eingestellt sein und handeln [16].

### Forschungsinfrastruktur ausbauen und Methoden weiterentwickeln.

Um diese Diversität besser zu erfassen, müssen Forschungsinfrastrukturen weiterentwickelt, verknüpft und ausgebaut werden. Wir benötigen bessere empirische Grundlagen sowohl für die Gruppe der Hochaltrigen generell als auch für unterschiedliche (häufig besonders schwer erreichbare und befragbare) Gruppen wie Personen mit Pflegebedarf oder verschiedener kultureller Herkunft – ein Desiderat insbesondere für (repräsentative) quantitative Studien. Erste Schritte in diese Richtung sind mit der Etablierung einer Hochaltrigkeitsstudie [70] in Deutschland getan – ihre Erweiterung und Fortführung sowie die bessere Verschränkung der Forschungsfelder „Alter(n)“ und „Umwelt“ bleiben aber eine Zukunftsaufgabe.

Weiterhin wissen wir noch zu wenig über die subjektiven Gestaltungsmöglichkeiten Älterer und insbesondere eben der Personengruppen in besonders vulnerablen Lebenslagen (Hochaltrige, Personen in Institutionen, mit gesundheitlichen Einschränkungen, Migrationshintergrund, Armut etc.) – eine Aufgabe vor allem für vertiefende qualitative Betrachtungen. Zur Entwicklung gezielter evidenzbasierter Maßnahmen zur Verbesserung der Lebenssituation(en) Älterer und Hochaltriger bedarf es letztlich gemischt-methodischer Studien, die ihre Bedarfe und Ressourcen a) zur Anpassung an die Herausforderungen an den Klimawandel wie auch b) zur aktiven Mitgestaltung der Reaktionen auf den Klimawandel analysieren.

## Fazit

Ältere und insbesondere Hochaltrige mit geringen sozioökonomischen Ressourcen und in der stationären Pflege sind gegenüber klimabedingten Risiken besonders vulnerabel. Trotz der Ausweitung und Zuspitzung dieser Problematik in Zeiten von rapider Alterung und Klimaveränderungen liegen bisher kaum Erkenntnisse an der Schnittstelle von Alter(n)sforschung und Umwelt- und Nachhaltigkeitsforschung vor, welche Folgen und Bedarfe dies nach sich zieht. Um das neu entstehende Forschungsfeld systematisch zu erschließen und diese Forschungslücken bearbeiten zu können, plädieren wir in diesem Beitrag für die gezielte Erweiterung von theoretischen Modellen, den Ausbau der Forschungsinfrastruktur und die Weiterentwicklung von Forschungsmethoden. Gerade in Zeiten des beschleunigten Wandels müssen wissenschaftliche Modelle intergenerationeller Umweltgerechtigkeit dringend weiterentwickelt und an aktuelle Herausforderungen angepasst werden – letztlich mit dem Ziel, sie auf Basis evidenzbasierter Maßnahmen gesellschaftlich zu etablieren.

## Data Availability

Alle dieser Arbeit zugrunde liegenden Daten sind in diesem Artikel enthalten.

## Footnotes

1 https://register.awmf.org/de/leitlinien/detail/053-061; zugegriffen am 27.04.2026.
